# Impact of Phosphoric Acid Etching Duration on the Bonding Performance of Universal Adhesives on Enamel: A Systematic Review of Laboratory Studies

**DOI:** 10.1111/jerd.70057

**Published:** 2025-11-26

**Authors:** Tarek Amran, Jeronim Esati, Roland Weiger, Markus B. Blatz, Florin Eggmann

**Affiliations:** ^1^ Department of Periodontology, Endodontology, and Cariology, University Center for Dental Medicine UZB University of Basel Basel Switzerland; ^2^ Department of Preventive and Restorative Sciences, School of Dental Medicine University of Pennsylvania Philadelphia Pennsylvania USA

**Keywords:** dental acid etching, dental bonding, dental materials, dental restoration failure, in vitro techniques, permanent dental restoration

## Abstract

**Objective:**

The optimal duration of phosphoric acid etching (PAE) for enamel bonding remains uncertain, particularly in the context of universal adhesives. The aim of this systematic review was to determine whether shortened etching times (< 15 s) provide comparable bond strength to conventional protocols.

**Materials and Methods:**

Searches were conducted across four databases, including Embase, OpenGrey through DANS, PubMed, and Scopus. Laboratory studies on human or bovine enamel specimens, treated with varying PAE durations prior to the application of universal adhesives, were included. Data regarding etching protocols, adhesive types, substrate preparation (ground vs. unground enamel), and bond strength outcomes were analyzed. Risk of bias was evaluated using the RoBDEMAT tool.

**Results:**

Of 762 records screened, eight laboratory studies met inclusion criteria. Etching times of 3–15 s achieved bond strengths that were statistically non‐inferior to conventional durations, with no added benefit from prolonged etching. Ground enamel consistently showed enhanced bond strength with PAE, whereas unground enamel exhibited similar bonding trends. The risk of bias was most often due to inadequate sample size justification, deficiencies in randomization, and absence of blinding.

**Conclusions:**

Short‐duration PAE (3–15 s) appears sufficient for effective bonding of universal adhesives to enamel, while minimizing potential risk of over‐etching of adjacent dentin. Future research should focus on tailoring etching protocols based on cavity‐specific characteristics and exploring alternative pretreatment methods to enhance bonding performance.

**Clinical Significance:**

This review suggests that abbreviated phosphoric acid etching (3–15 s) provides enamel bond strengths equivalent to conventional durations when universal adhesives are used. This approach may reduce the risk of inadvertent dentin over‐etching in mixed‐substrate cavities while maintaining optimal adhesive performance.

## 1 Introduction

Phosphoric acid etching (PAE) is widely recognized for enhancing enamel bond strength, regardless of the type of dental adhesive subsequently applied [1, 2, 3, 4, 5]. Etch‐and‐rinse adhesives rely on PAE of both enamel and dentin, with enamel typically requiring longer etching durations than dentin to achieve adequate bonding [6]. Conversely, self‐etch adhesives require that PAE on dentin be avoided, as it can compromise bonding effectiveness [7, 8].

The introduction of universal adhesives has made it optional to etch dentin with phosphoric acid, allowing dental practitioners to opt for either an etch‐and‐rinse approach or a selective enamel etching technique [1, 9, 10]. However, selective enamel etching presents clinical challenges. Precise confinement of the etchant to enamel can be difficult, especially in proximal cavities and when the dentin‐enamel junction is hard to discern [11, 12, 13]. Computer‐assisted imaging analysis has demonstrated that dental practitioners frequently—and inadvertently—extend the etchant onto dentin, thereby increasing the risk of over‐etching [11]. Such over‐etching may compromise bond integrity and potentially reduce the long‐term durability of adhesive restorations [8].

To mitigate the risk of inadvertent dentin over‐etching, reducing PAE duration on enamel has been proposed as a pragmatic clinical strategy [11, 14]. Recent laboratory studies have investigated whether shorter PAE durations affect the bonding performance of universal adhesives [14, 15, 16, 17, 18, 19, 20, 21, 22]. Given the critical role of PAE in achieving durable adhesion, a systematic evaluation of laboratory evidence is necessary to better understand how variations in etching duration influence bonding outcomes.

This systematic review aims to synthesize existing laboratory studies on the impact of PAE duration on the bonding performance of universal adhesives on enamel. The objectives are to determine whether reducing PAE duration compromises adhesive performance and to provide guidance for optimizing etching protocols in clinical practice.

## 2 Methods

### 2.1 Research Question

The protocol for this systematic review was prospectively registered in the International Prospective Register of Systematic Reviews (PROSPERO reference number [CRD42024503210]). This review adhered to the Preferred Reporting Items for Systematic Reviews and Meta‐Analyses (PRISMA) guidelines [23]. The research question was structured using the PICO (population, intervention, comparison, and outcome) framework: What is the impact of varying PAE durations, both shorter and longer than manufacturer‐recommended times, on the adhesive performance of universal adhesives to enamel in in vitro settings?

- *Population*: in vitro specimens featuring human or bovine enamel
- *Intervention*: enamel acid etching with phosphoric acid for varied durations (prolonged, shortened, or both) prior to the application of a universal adhesive
- *Comparison*: omission of enamel acid etching or standard enamel acid etching with phosphoric acid as per manufacturer recommendations prior to the application of a universal adhesive
- *Outcome*: adhesive performance, assessed through shear bond strength, tensile bond strength, or interfacial fracture toughness testing

### 2.2 Eligibility Criteria

Studies were selected based on the predefined inclusion and exclusion criteria detailed below. Only comprehensive study reports were considered for inclusion, with no restrictions on publication date or language.

### 2.3 Inclusion Criteria

Studies were included if they met the following criteria:

- In vitro/laboratory study
- Specimens made from or featuring human or bovine enamel
- Use of PAE on enamel with different application times
- Application of a universal adhesive
- Data on adhesive performance in terms of bond strength or interfacial fracture toughness

### 2.4 Exclusion Criteria

Studies were excluded if they met any of the following criteria:

- Abstract‐only paper
- Animal study
- Case Report
- Clinical study
- *In silico* study
- Poster
- Review article

### 2.5 Search Strategy

A systematic electronic search was conducted on November 3, 2025, encompassing four databases: Embase, OpenGrey (accessed through DANS), PubMed, and Scopus. Database‐specific search strategies incorporating controlled vocabulary terms and Boolean operators were developed and are provided in Table A1 (Supporting Information Online). To maximize search sensitivity, two investigators (T.A., J.E.) independently performed manual backward citation searching of all eligible studies, reviewing reference lists to identify potentially relevant articles not retrieved through database searches.

### 2.6 Selection Process

After the removal of duplicates, two investigators (T.A., J.E.) independently screened the titles and abstracts of the articles retrieved through the electronic search, applying the eligibility criteria to identify potentially relevant studies. During this process, author names and journals remained unblinded. Full‐text articles of potentially relevant studies were then retrieved for further assessment. The same investigators independently evaluated each study based on the eligibility criteria. Any disagreements regarding study eligibility were resolved through consultation with a third investigator (F.E.). When multiple reports pertained to the same study, they were linked and treated as a single record. Reasons for exclusion were documented.

### 2.7 Data Collection

Two investigators (T.A., J.E.) independently extracted both qualitative and quantitative data from the included studies using a pilot‐tested, structured spreadsheet. In cases of discrepancies, a third investigator (F.E.) reviewed the data and made the final decision.

### 2.8 Data Items

Data were extracted on the following variables: number of specimens, specimen type, pretreatment regimen, adhesive and restorative materials used, storage and aging conditions, bond strength assessment methods, and main findings (Table 1). “Comparable bond strength” was operationally defined as the absence of statistically significant differences (*p* > 0.05) between shortened and conventional etching protocols within individual studies.

**TABLE 1:** Overview of the main characteristics of laboratory studies included in the systematic review.

Study	Number of specimens	Specimens	Pretreatment regimen	Adhesive and restorative material	Storage/aging	Bond strength assessment	Main findings
Hamdy et al. [15] and Hamdy et al. [22]	96 human primary molar halves total. Phase I: 64 tooth halves across nine subgroups (*n* = 6–7). Phase II: 36 tooth halves across six subgroups (*n* = 7–8). Multiple sticks per tooth half tested (total: 1059 sticks).	Human enamel (deciduous, ground, sound)	Subgroup 1 (control): SE‐mode Subgroup 2: 15 s PAE (36%) Subgroup 3: 30 s PAE (36%)	Group 1: Scotchbond Universal Adhesive (3M oral care, Seefeld, Germany) Group 2: Clearfil Universal Bond Quick (Kuraray Noritake Dental, Okayama, Japan) Group 3: iBond Universal Adhesive (Heraeus Kulzer, Hanau, Germany) Restorative material in all groups: Filtek Z250 Universal Restorative System (3M Oral Care, Seefeld, Germany)	Phase I: Specimens were stored for 24 h at 37°C in distilled water prior to testing. Phase II: Specimens were stored for 6 months at 37°C in distilled water prior to testing.	Microtensile bond strength testing	PAE significantly enhanced bond strength to deciduous enamel in all tested groups. There were no significant differences between groups etched for 15 and 30 s. 6‐month aging significantly reduced bond strength only for Scotchbond Universal Adhesive with 15 s etching.
Wong et al. [16]	Insufficiently reported	Human enamel (permanent, ground)	Control group: no pretreatment Subgroup 1: PAE (35%) Subgroup 2: polyalkenoic acid etching Subgroup 3: phosphoric acid ester monomer etching Sub‐subgroup 1: less than 1 s Sub‐subgroup 2: 5 s Sub‐subgroup 3: 10 s Sub‐subgroup 4: 15 s	Group 1: G‐Premio Bond (GC, Tokyo, Japan) Group 2: Prime&Bond Elect (Dentsply Sirona, Milford, DE, USA) Group 3: Scotchbond Universal Adhesive (3M Oral Care, St Paul, MN, USA) Restorative material in all groups: Z100 Restorative (3M Oral Care, St Paul, MN, USA)	Specimens were stored in 37°C distilled water for 24 h prior to initial bond strength testing. The bonded fatigue specimens were loaded using a sine wave at a frequency of 20 Hz for 50,000 cycles or until failure occurred with a staircase method.	Initial and fatigue shear bond strength testing	PAE for less than 1–15 s improved bond strength of universal adhesives.
Shafiei et al. [17]	84 in total, seven groups (*n* = 12)	Human enamel (permanent, unground, sound)	Group 1 (control): 30 s PAE (*) Group 2: 10 s Er, Cr:YSGG laser etching Group 3: 15 s PAE (*) Group 4: 10 s PAE (*) Group 5: 5 s PAE (*) Group 6: SE‐mode Group 7: 10 s Er, Cr:YSGG laser etching *Percentage not specified	Group 1: Transbond XT Primer (3M Oral Care, St Paul, MN, USA) Group 2: Transbond XT Primer (3M Oral Care, St Paul, MN, USA) Group 3: Scotchbond Universal Adhesive (3M Oral Care, St Paul, MN, USA) Group 4: Scotchbond Universal Adhesive (3M Oral Care, St Paul, MN, USA) Group 5: Scotchbond Universal Adhesive (3M Oral Care, St Paul, MN, USA) Group 6: Scotchbond Universal Adhesive (3M Oral Care, St Paul, MN, USA) Group 7: Scotchbond Universal Adhesive (3M Oral Care, St Paul, MN, USA) Restorative material in all groups: Transbond XT (3M Oral Care, St Paul, MN, USA)	Specimens were stored in 37°C distilled water and then thermocycled for 3000 cycles between 5°C and 55°C with a dwell time of 30 s.	Shear bond strength testing	PAE improved shear bond strength. However, increasing the duration from 5 to 15 s did not result in significantly different shear bond strengths, while causing enamel damage in the form of cracks.
Shimatani et al. [14]	1170 in total, three groups, 78 sub‐subgroups (*n* = 15)	Bovine enamel (permanent, ground)	Control group: no pretreatment Subgroup 1: phosphoric acid ester monomer etching Subgroup 2: PAE (35%) Subgroup 3: polyalkenoic acid etching Sub‐subgroup 1: less than 1 s Sub‐subgroup 2: 5 s Sub‐subgroup 3: 10 s Sub‐subgroup 4: 15 s	Group 1: BeautiBond Universal (Shofu, Kyoto, Japan) Group 2: Prime & Bond Elect (Dentsply Sirona, Milford, DE, USA) Group 3: Scotchbond Universal Adhesive (3M Oral Care, St Paul, MN, USA) Restorative material in all groups: Filtek Supreme Ultra Restorative (3M Oral Care, St Paul, MN, USA)	Specimens were stored in 37°C distilled water for 24 h. Each specimen was then allocated randomly to one of two groups with different storage conditions: either no thermal cycling or 10,000 thermal cycles between 5°C and 60°C.	Shear bond strength testing	Enamel bonding with universal adhesives was improved by reduced PAE times of less than 1–15 s, with no significant differences between etching durations.
Tsujimoto et al. [18]	Initial shear shear bond strength testing: 225 in total, three groups, 15 subgroups (*n* = 15) Fatigue shear bond strength testing: 300 in total, three groups, 15 subgroups (*n* = 20)	Human enamel (permanent, ground)	Subgroup 1 (control group): no pretreatment Subgroup 2: less than 1 s PAE (35%) Subgroup 3: 5 s PAE (35%) Subgroup 4: 10 s PAE (35%) Subgroup 5: 15 s PAE (35%)	Group 1: Clearfil Universal Bond Quick (Kuraray Noritake, Tokyo, Japan) Group 2: G‐Premio Bond (GC, Tokyo, Japan) Group 3: Scotchbond Universal Adhesive (3M ESPE, St Paul, MN, USA) Restorative material in all groups: Z100 Restorative (3M ESPE, St Paul, MN, USA)	Specimens were stored in 37°C distilled water for 24 h prior to testing.	Initial and fatigue shear bond strength testing	PAE, regardless of application times—ranging from less than 1 to 15 s, improved initial and fatigue bond strength.
Tsujimoto et al. [19]	Three groups, 18 subgroups, 54 sub‐subgroups (*n* = 15)	Human enamel (permanent, ground, sound)	Subgroup 1: SE‐mode, smear layer not removed Subgroup 2: SE‐mode, smear layer removed by ultrasonic cleaning Subgroup 3: 3 s PAE (35%) Subgroup 4: 5 s PAE (35%) Subgroup 5: 10 s PAE (35%) Subgroup 6: 15 s PAE (35%)	Group 1: Scotchbond Universal Adhesive (3M ESPE, St Paul, MN, USA) Group 2: Adhese Universal (Ivoclar Vivadent, Schaan, Lichtenstein) Group 3: G‐Premio Bond (GC, Tokyo, Japan) Restorative material in all groups: Z100 Restorative (3M ESPE, St Paul, MN, USA)	Sub‐subgroup 1: 24 h in 37°C distilled water Sub‐subgroup 2: 30,000 thermal cycles between 5°C and 60°C Sub‐subgroup 3: 60,000 thermal cycles between 5°C and 60°C	Shear bond strength testing	PAE of enamel improves the bond durability of universal adhesives and the surface free‐energy characteristics of enamel, but these bonding properties do not increase for PAE times of longer than 3 s.
Takamizawa et al. [20]	Initial shear bond strength testing: 240 in total, four groups, 16 subgroups (*n* = 15) Fatigue shear bond strength testing: 480 in total, four groups, 16 subgroups (*n* = 30)	Human enamel (permanent, ground, sound)	Subgroup 1 (control group): no pretreatment Subgroup 2: 3 s PAE (35%) Subgroup 3: 10 s PAE (35%) Subgroup 4: 15 s PAE (35%)	Group 1: Prime&Bond Elect (Dentsply Caulk, Milford, DE, USA) Group 2: Scotchbond Universal Adhesive (3M ESPE, St Paul, MN USA) Group 3: G‐ӕnial Bond (GC, Tokyo, Japan) Group 4: OptiBond XTR (Kerr, Orange, CA, USA) Restorative material in all groups: Z100 Restorative (3M ESPE, St Paul, MN, USA)	Specimens were stored in 37°C distilled water for 24 h prior to testing.	Initial and fatigue shear bond strength test	PAE, with application durations ranging from 3 to 15 s, enhanced both initial and fatigue bond strength. However, PAE times longer than 3 s did not further improve bond strength to a significant degree.
Tsujimoto et al. [21]	270 in total, three groups, 18 subgroups (*n* = 15)	Bovine enamel (permanent, ground, sound)	Subgroup 1: SE‐mode, smear layer not removed Subgroup 2: SE‐mode, smear layer removed by ultrasonic cleaning Subgroup 3: 3 s PAE (37%) Subgroup 4: 5 s PAE (37%) Subgroup 5: 10 s PAE (37%) Subgroup 6: 15 s PAE (37%)	Group 1: Scotchbond Universal Adhesive (3M ESPE, St Paul, MN, USA) Group 2: Clearfil tri‐S Bond (Kuraray Noritake, Tokyo, Japan) Group 3: G‐Bond Plus (GC, Tokyo, Japan) Restorative material in all groups: Clearfil AP‐X (Kuraray Noritake, Tokyo, Japan)	Specimens were stored in 37°C distilled water for 24 h prior to testing.	Shear bond strength testing	PAE increased the enamel shear bond strength, but durations over 3 s did not increase it further. There were no significant differences in bond strength between the specimens with and without smear layer.

### 2.9 Study Risk of Bias Evaluation

Two investigators (T.A., J.E.) independently evaluated the risk of bias in the included studies using the RoBDEMAT tool [24]. A separate RoBDEMAT assessment was completed for each study (Table 2). Any discrepancies between assessments were resolved by a third investigator (F.E.).

**TABLE 2:** Results of the risk of bias assessment. Each RoBDEMAT signaling question in the four domains, D1, D2, D3, and D4, was answered as either “sufficiently reported/adequate” (1), “insufficiently reported” (2), “not reported/not adequate” (3), or “not applicable” (4).

Study	D1			D2		D3		D4	
	(1.1) Control group	(1.2) Randomization of samples	(1.3) Sample size rationale and reporting	(2.1) Standardization of samples and materials	(2.2) Identical experimental conditions across groups	(3.1) Adequate and standardized testing procedures and outcomes	(3.2) Blinding of the test operator	(4.1) Statistical analysis	(4.2) Reporting study outcomes
Hamdy et al. [15] and Hamdy et al. [22]	1	2	1	1	1	1	3	1	1
Wong et al. [16]	1	3	3	1	1	1	3	1	1
Shafiei et al. [17]	1	2	3	2	2	1	3	2	1
Shimatani et al. [14]	1	2	3	1	1	1	3	1	1
Tsujimoto et al. [18]	1	3	3	2	1	1	3	1	1
Tsujimoto et al. [19]	1	2	3	1	1	1	3	1	1
Takamizawa et al. [20]	1	3	3	1	2	1	3	1	1
Tsujimoto et al. [21]	1	3	3	1	2	1	3	1	1

## 3 Results

### 3.1 Included Studies

**FIGURE 1:**
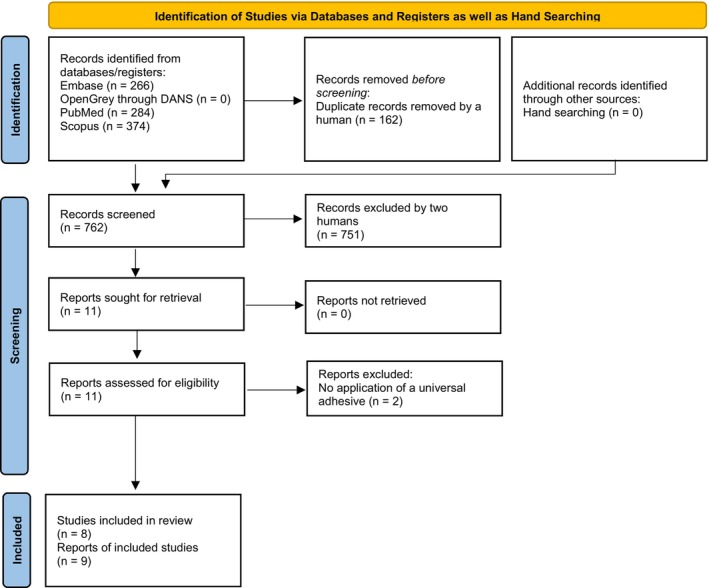
PRISMA 2020 flow diagram depicting the selection of records for this review [23].

Figure 1 illustrates the study selection process, which resulted in the inclusion of eight laboratory studies (reported across nine publications) published between 2016 and 2025 [14, 15, 16, 17, 18, 19, 20, 21, 22]. Detailed characteristics and outcomes of these studies are presented in Table 1. Two full‐text articles were excluded at the eligibility stage owing to the absence of universal adhesive evaluation in their protocols [25, 26].

### 3.2 Characteristics of Included Studies

#### 3.2.1 Specimens and Materials

The included studies utilized both human enamel—deciduous and permanent—and bovine enamel as bonding substrates. The most commonly tested universal adhesive was Scotchbond Universal Adhesive (3M Oral Care, St Paul, MN, USA) [14, 15, 16, 17, 18, 19, 20, 21, 22]. Other adhesives evaluated were Adhese Universal (Ivoclar Vivadent, Schaan, Lichtenstein) [19], BeautiBond Universal (Shofu, Kyoto, Japan) [14], Clearfil Universal Bond Quick (Kuraray Noritake, Tokyo, Japan) [15, 18], G‐Premio Bond (GC, Tokyo, Japan) [16, 18, 19], iBond Universal Adhesive (Heraeus Kulzer, Hanau, Germany) [15], and Prime&Bond Elect (Dentsply Sirona, Milford, DE, USA) [14, 16, 20]. All restorative materials used were resin‐based composites, namely Clearfil AP‐X (Kuraray Noritake, Tokyo, Japan) [21], Filtek Supreme Ultra Restorative (3M Oral Care, St Paul, MN, USA) [14], Filtek Z250 Universal Restorative System (3M Oral Care, Seefeld, Germany) [15, 22], Transbond XT (3M Oral Care, St Paul, MN, USA) [17], Z100 Restorative (3M ESPE, St Paul, MN, USA) [16, 18, 19, 20].

#### 3.2.2 Pretreatment Regimens

PAE durations varied across studies, ranging from less than 1–30 s. Concentrations of the phosphoric acid etchants were typically around 35%–37%.

#### 3.2.3 Storage and Aging

Most specimens were stored in 37°C distilled water for 24 h prior to bond strength testing. Three studies included additional aging protocols, such as thermocycling, by first storing specimens in 37°C distilled water followed by thermocycling [14, 17, 19]. The number of thermocycles ranged from 3000 to 600,000 cycles. Two studies used both non‐thermocycled and thermocycled conditions [14, 19]. One study assessed the effects of water storage for 24 h and 6 months [22].

#### 3.2.4 Bond Strength Assessments

Most studies used shear bond strength testing to assess the bond strength of the adhesives to enamel. Three studies tested initial and fatigue shear bond strength [16, 18, 20]. One study employed microtensile bond strength testing [15, 22].

#### 3.2.5 Effects on Enamel Bond Strength

Across all included studies, statistical comparisons between shortened (3–15 s) and conventional (15–30 s) PAE durations consistently demonstrated non‐significant differences (*p* > 0.05), indicating equivalent bonding performance. Meta‐analysis was not feasible owing to substantial heterogeneity in study designs, substrates, adhesive systems, and testing methodologies.

#### 3.2.6 Permanent Versus Deciduous Teeth

This systematic review included mostly studies on human permanent enamel, with one study that investigated deciduous enamel [15, 22]. In terms of the effect of PAE on bond strength, both permanent and deciduous enamel showed a significant improvement after etching when using universal adhesives [14, 15, 16, 17, 18, 19, 20, 22]. Regarding etching duration, studies on permanent enamel demonstrated that etching times between 3 and 15 s produced similar improvements in bond strength, with no significant additional benefit from longer application times [14, 16, 17, 18, 19, 20]. Similarly, the study on deciduous enamel also reported no significant difference between 15 and 30 s etching durations [22].

#### 3.2.7 Human Versus Bovine Enamel

This systematic review included two studies that utilized bovine enamel, both of which demonstrated that shortened phosphoric acid etching durations improved bond strength [14, 21]. Similarly, six studies conducted on human enamel showed consistent improvements in bond strength with PAE, even with reduced etching times [14, 15, 16, 17, 18, 19, 22]. However, no study directly compared bond strength outcomes between human and bovine enamel.

#### 3.2.8 Ground Versus Unground Enamel

Most studies used ground enamel, finding consistent improvements in bond strength with PAE pretreatment [14, 15, 16, 18, 19, 20, 21, 22]. One investigation used unground enamel specimens, demonstrating similar improvements in bond strength with PAE pretreatment [17]. Increasing etching duration from 5 to 15 s did not significantly affect bond strength but caused enamel damage in the form of cracks [17].

### 3.3 Risk of Bias

Table 2 provides detailed RoBDEMAT assessments of the included studies. Most studies performed well in standardizing procedures and reporting statistical analyses. Common issues identified were sample size justification, randomization of specimens, and blinding.

## 4 Discussion

This systematic review evaluated laboratory evidence on the influence of PAE duration on the bonding performance of universal adhesives to enamel. Eight in vitro studies published between 2016 and 2025 were included, examining various universal adhesives applied to human or bovine enamel under different PAE protocols. The primary goal was to determine whether shorter etching times, typically under 15 s, could achieve bond strengths comparable to conventional durations of 15–30 s. The findings consistently indicated that shorter PAE durations were sufficient to optimize enamel bonding, with no added benefit observed from extended etching times [14, 15, 16, 17, 18, 19, 20, 21, 22].

Most of the reviewed studies employed ground enamel specimens, which provide a more uniform and controlled surface for bonding. Ground enamel showed consistent improvements in bond strength with PAE pretreatment. Comparable results were also observed in unground enamel [17]. This finding holds clinical relevance for situations involving unprepared enamel surfaces, such as fissure sealants, peg‐shaped lateral incisors, diastema closure, or orthodontic bracket bonding. However, as only one study has specifically examined this approach in unground enamel, further research is warranted to confirm its effectiveness. In cases where both enamel and dentin are present, reducing etching time may help mitigate the risk of dentin overetching while maintaining adequate enamel conditioning [11]. By contrast, in restorations confined to unground enamel, where dentin is not involved, the advantages of shortened PAE appear less pronounced and may confer little, if any, clinical benefit.

The role of the smear layer in enamel bonding with universal adhesives has been examined in studies highlighting that even brief PAE effectively removes the smear layer, enabling uniform resin infiltration and yielding stable, high bond strength [18, 19]. Notably, longer PAE durations offer no additional benefit, suggesting that minimal etching is sufficient to optimize the enamel bonding interface.

In addition to PAE, some studies explored alternative enamel pretreatment methods, including laser etching and polyalkenoic acid etching. Comparisons between laser etching and PAE revealed no significant differences in bond strength, suggesting that both techniques may be similarly effective in preparing the enamel surface [17]. Polyalkenoic acid, a milder etchant commonly used for surface conditioning in glass ionomer restorations, was also evaluated [14, 16]. In a study assessing various application times, a 15‐s polyalkenoic acid pretreatment yielded bond strengths equivalent to those achieved with PAE, while shorter durations were less effective [16]. Though these findings suggest that polyalkenoic acid may serve as a viable alternative under certain conditions, further laboratory and clinical studies are needed to establish its effectiveness as a pretreatment for universal adhesive bonding to enamel.

As new adhesives are introduced and existing products undergo refinement, their performance should be systematically evaluated under varying etching protocols to determine optimal application strategies. This systematic review suggests that shortened PAE times are sufficient to achieve effective enamel bonding with universal adhesives. However, the effectiveness of these adhesives depends not only on etching time but also on their chemical composition and interaction with enamel substrates [10]. Recent experimental evidence has shown that adhesives incorporating carboxylic acid‐based monomers tend to cause greater enamel demineralization and surface roughening, whereas those containing phosphate‐based functional monomers, such as glycerol phosphate dimethacrylate and 10‐methacryloyloxy‐decyl‐di‐hydrogenphosphate, demonstrate enhanced wettability and superior bond strengths [10]. These findings underscore the importance of informed product selection and protocol tailoring, emphasizing that both the chemical formulation of the adhesive and its clinical application strategy must be aligned to optimize outcomes in adhesive dentistry.

To assess the methodological robustness of the included studies, the risk of bias was evaluated using the RoBDEMAT tool [24]. Most studies demonstrated strong standardization of procedures and thorough reporting of statistical analyses. However, common issues were identified, such as insufficient justification for sample size, inadequate randomization, and a lack of blinding. Addressing these limitations in future research will be crucial to enhancing the reliability and validity of outcomes in this field.

Several limitations of this systematic review warrant consideration [27]. The substantial heterogeneity in methodologies, materials, and testing protocols across studies precluded quantitative synthesis through meta‐analysis and limits the generalizability of findings. Furthermore, all studies were conducted under in vitro conditions, which, while enabling precise control of variables such as PAE duration and systematic assessment of bond strength, cannot fully replicate the complexity of the clinical environment. In vivo, numerous factors—including saliva composition and flow, masticatory forces, thermal cycling, long‐term hydrolytic degradation, and patient‐specific variables such as age, oral hygiene practices, dietary habits, and inherent variations in enamel structure—can substantially influence bonding performance and restoration longevity. Consequently, while laboratory studies provide essential mechanistic insights into adhesive behavior, they offer limited predictive value for clinical outcomes. Randomized controlled clinical trials are therefore essential to validate these in vitro findings and establish their clinical relevance [24]. Additionally, data regarding the effects of storage duration and aging conditions on bond strength remain limited. Some evidence suggests that shorter PAE durations may compromise the long‐term bond durability of certain universal adhesives to enamel of deciduous teeth, underscoring the need for cautious interpretation of immediate bond strength values and emphasizing the importance of aging studies in future research [22].

Given these limitations, only tentative clinical recommendations can be formulated. Current evidence suggests that universal adhesives applied following PAE for approximately 3–15 s may provide adequate enamel bond strength in routine restorative procedures. In mixed‐substrate cavities involving both enamel and dentin, prolonged etching should be avoided, as it may compromise dentin bonding performance. Dental practitioners should exercise particular care during selective enamel etching in such scenarios, as inadvertent over‐etching of adjacent dentin represents a risk that can adversely affect restoration integrity.

From a clinical perspective, the practical implications of PAE timing warrant consideration in the context of large preparations such as extensive mesio‐occluso‐distal cavities. In these cases, the etchant is typically applied sequentially along the enamel margins, creating an inherent gradient in etching duration: areas contacted first receive longer exposure, while subsequently treated regions undergo correspondingly shorter etching times. The present findings—demonstrating that abbreviated PAE durations achieve bond strengths comparable to conventional etching times—provide reassurance that this sequential application approach does not compromise bonding effectiveness when universal adhesives are employed.

These results support a pragmatic clinical protocol: dental practitioners should initiate timing at the moment of initial etchant contact with enamel, recognizing that subsequently treated areas will inherently receive shorter exposure. Rather than representing a limitation, this variation in etching duration falls within an acceptable range that maintains adequate bond strength across all cavity margins.

## 5 Conclusion

Within the constraints of this systematic review of laboratory studies, the following conclusions can be drawn:

- *Etching duration*: Current in vitro evidence indicates that PAE durations of 3–15 s achieve enamel bond strengths comparable to conventional etching times (15–30 s), with no significant benefit from prolonged etching when universal adhesives are used.
- *Clinical implications*: Abbreviated PAE protocols (3–15 s) appear clinically acceptable for selective enamel etching with universal adhesives, offering dual advantages: maintaining adequate bond strength while reducing the risk of inadvertent dentin over‐etching in mixed‐substrate preparations.
- *Future research*: Clinical studies are needed to further validate the effectiveness of shorter PAE durations. Additionally, research should focus on developing tailored etching protocols based on cavity‐specific characteristics and exploring alternative pretreatment methods to optimize clinical outcomes in adhesive dentistry.

## Conflicts of Interest

The authors declare no conflicts of interest.

## Supporting information


**Data S1:** Supporting Information.

## Data Availability

All data supporting the findings of this article are fully presented in the main text and the accompanying Supplementary File.
