# Supplementary material for: Impact of Phosphoric Acid Etching Duration on the Bonding Performance of Universal Adhesives on Enamel: A Systematic Review of Laboratory Studies
Source: J Esthet Restor Dent. 2025 Nov 26;38(4):780–91. doi: 10.1111/jerd.70057 (PMC13069211; doi:10.1111/jerd.70057)
Supplement: Supplementary file 1 — Data S1: Supporting Information. [file JERD-38-780-s001.pdf]

Impact of Phosphoric Acid Etching Duration on the Bonding Performance of Universal Adhesives on Enamel: A Systematic Review of Laboratory Studies

Appendix

Table A1 Information on the query strings

Database	Query string
Embase	('enamel'/exp OR 'enamel':ab,ti OR 'dental enamel'/exp OR 'dental enamel':ab,ti OR 'tooth surface*':ab,ti) AND ('etch*':ab,ti OR 'etch-and-rinse':ab,ti OR 'condition*':ab,ti OR 'phosphoric acid*':ab,ti OR 'phosphoric acids'/exp OR 'phosphoric acids':ab,ti OR 'dental etching'/exp OR 'dental etching':ab,ti OR 'acid etching, dental'/exp OR 'acid etching, dental':ab,ti OR 'orthophosphoric acid*':ab,ti OR 'H3PO4':ab,ti) AND ('bond strength*':ab,ti OR 'bond-strength*':ab,ti OR 'adhesion*':ab,ti OR 'adhesive performance*':ab,ti OR 'bond performance*':ab,ti OR 'bonding performance*':ab,ti OR 'interfacial fracture toughness':ab,ti OR 'bonding effectiveness':ab,ti OR 'adhesive effectiveness':ab,ti OR 'shear fatigue strength*':ab,ti OR 'shear strength*':ab,ti OR 'tensile fatigue strength*':ab,ti OR 'tensile strength*':ab,ti OR 'enamel bond*':ab,ti) AND ('universal adhesive*':ab,ti OR 'multi-mode adhesive*':ab,ti OR 'multimode adhesive*':ab,ti OR 'multimode*':ab,ti OR 'multi mode adhesive*':ab,ti OR 'universal bond*':ab,ti OR 'simplified adhesive*':ab,ti OR 'multipurpose adhesive*':ab,ti OR 'multi-purpose adhesive*':ab,ti OR 'multi purpose adhesive*':ab,ti OR 'all in one':ab,ti OR 'all-in-one':ab,ti OR 'universal self etching*':ab,ti OR 'universal self-etching*':ab,ti)
OpenGrey through DANS	(enamel OR "Dental Enamel" OR "tooth surface*") AND ("etch*" OR "etch-and-rinse" OR "condition*" OR "phosphoric acid*" OR "Phosphoric Acids" OR "Dental Etching" OR "Acid Etching, Dental" OR "orthophosphoric acid*" OR "H3PO4") AND ("bond strength*" OR "bond-strength*" OR "adhesion*" OR "adhesive performance*" OR "bond performance*" OR "bonding performance*" OR "interfacial fracture toughness" OR "bonding effectiveness" OR "adhesive effectiveness" OR "shear fatigue strength*" OR "shear strength*" OR "tensile fatigue strength*" OR "tensile strength*" OR "enamel bond*") AND ("universal adhesive*" OR "multi-mode adhesive*" OR "multimode adhesive*" OR "multimode*" OR "multi mode adhesive*" OR "universal bond*" OR "simplified adhesive*" OR "multipurpose adhesive*" OR "multi-purpose adhesive*" OR "multi purpose adhesive*" OR "'multi-mode' adhesive*" OR "all in one" OR "all-in-one" OR "universal self etching*" OR "universal self-etching*")
PubMed	("enamel"[tiab] OR "Dental Enamel"[mesh] OR "tooth surface*"[tiab]) AND ("etch*"[tiab] OR "etch-and-rinse"[tiab] OR "condition*"[tiab] OR "phosphoric acid*"[tiab] OR "Phosphoric Acids"[mesh] OR "Dental Etching"[mesh] OR

	"Acid Etching, Dental"[mesh] OR “orthophosphoric acid*”[tiab] OR “H3PO4”[tiab]) AND (“bond strength*”[tiab] OR “bond-strength*”[tiab] OR “adhesion*”[tiab] OR “adhesive performance*”[tiab] OR “bond performance*”[tiab] OR “bonding performance*”[tiab] OR “interfacial fracture toughness”[tiab] OR “bonding effectiveness”[tiab] OR “adhesive effectiveness”[tiab] OR “shear fatigue strength*”[tiab] OR “shear strength*”[tiab] OR “tensile fatigue strength*”[tiab] OR “tensile strength*”[tiab] OR “enamel bond*”[tiab]) AND (“universal adhesive*”[tiab] OR “multi-mode adhesive*”[tiab] OR “multimode adhesive*”[tiab] OR “multimode*”[tiab]OR “multi mode adhesive*”[tiab] OR “universal bond*”[tiab] OR “simplified adhesive*”[tiab] OR “multipurpose adhesive*”[tiab] OR “multi-purpose adhesive*”[tiab] OR “multi purpose adhesive*”[tiab] OR “‘multi-mode’ adhesive*”[tiab] OR “all in one”[tiab] OR “all-in-one”[tiab] OR “universal self etching*”[tiab] OR “universal self-etching*”[tiab])
Scopus	(TITLE-ABS-KEY (enamel OR "Dental Enamel" OR “tooth surface*”) AND TITLE-ABS-KEY ("etch*" OR “etch-and-rinse” OR “condition*” OR “phosphoric acid*” OR "Phosphoric Acids" OR "Dental Etching" OR "Acid Etching, Dental" OR “orthophosphoric acid*” OR “H3PO4”)) AND (TITLE-ABS-KEY (“bond strength*” OR “bond-strength*” OR “adhesion*” OR “adhesive performance*” OR “bond performance*” OR “bonding performance*” OR “interfacial fracture toughness” OR “bonding effectiveness” OR “adhesive effectiveness” OR “shear fatigue strength*” OR “shear strength*” OR “tensile fatigue strength*” OR “tensile strength*” OR “enamel bond*”)) AND (TITLE-ABS-KEY (“universal adhesive*” OR “multi-mode adhesive*” OR “multimode adhesive*” OR “multimode*” OR “multi mode adhesive*” OR “universal bond*” OR “simplified adhesive*” OR “multipurpose adhesive*” OR “multi-purpose adhesive*” OR “multi purpose adhesive*” OR “‘multi-mode’ adhesive*” OR “all in one” OR “all-in-one” OR “universal self etching*” OR “universal self-etching*”))
